# Label-free ultraviolet photoacoustic microscopy for multiscale structural phenotyping of epithelial morphogenesis in organoid-derived intestinal epithelial wrinkle tissue

**DOI:** 10.1016/j.pacs.2026.100875

**Published:** 2026-08-24

**Authors:** Linbin Zha, Jangwon Yoon, Santanu Misra, Hyunjun Kye, Jeesu Kim, Jaesung Youn, Dong Sung Kim, Byullee Park

**Affiliations:** a Department of Biophysics, Department of MetaBioHealth, Department of Biopharmaceutical Convergence, Institute of Quantum Biophysics, Sungkyunkwan University, Suwon, Republic of Korea; b Department of Mechanical Engineering, Pohang University of Science and Technology (POSTECH), Pohang, Republic of Korea; c Department of Cogno-Mechatronics Engineering, Pusan National University, Busan 46241, Republic of Korea; d Department of Medical Science, School of Medicine, CHA University, Seongnam, Republic of Korea; e Graduate School of Convergence Science and Technology, Pohang University of Science and Technology (POSTECH), Pohang, Republic of Korea; f Division of Interdisciplinary Bioscience & Bioengineering, Pohang University of Science and Technology (POSTECH), Pohang, Republic of Korea

**Keywords:** Ultraviolet photoacoustic microscopy, Organoid-derived epithelial wrinkle tissue, Epithelial morphogenesis, Deep-learning classification

## Abstract

Mechanical wrinkling of epithelial tissues encodes key features of tissue morphogenesis and mechanotransductive responses, yet its quantitative, multiscale characterization remains challenging in organoid-based in vitro models due to reliance on fluorescence labelling and sectioning. Here, we develop label-free ultraviolet photoacoustic microscopy (UV-PAM) for multiscale imaging and quantitative analysis of human organoid-derived epithelial wrinkle tissues. By exploiting intrinsic nucleic-acid absorption at 266 nm, UV-PAM visualizes wrinkle structures and nuclear morphology without labeling or sectioning. Using morphomechanically defined wrinkle tissues, we identify multiscale signatures, including wrinkle wavelength, wrinkle index, and strain-induced nuclear elongation and redistribution. UV-PAM further captures chemically induced epithelial stress, revealing degradation of wrinkle structure and nuclear morphology. In a proof-of-concept dataset comprising two untreated and two DTT-treated specimens, integration of these intrinsic signatures with deep learning enabled patch-level discrimination between untreated and chemically perturbed wrinkle tissues on held-out field of views (FOVs), with training and test FOVs obtained from the same specimens. These results establish UV-PAM as a label-free platform for multiscale imaging of epithelial morphogenesis and preliminary computational phenotyping.

## 1 Introduction

Mechanical wrinkling driven by compressive strain is a fundamental morphogenetic program in epithelial tissues, governing tissue structure, mechanical compliance, and functional organization across multiple length scales [1], [2], [3], [4]. At the tissue level, wrinkle structures accommodate large deformations while preserving structural integrity, whereas at the cellular and subcellular levels, compression-induced deformation of cells and nuclei regulates mechanotransduction, gene expression, and collective cellular behavior [5], [6], [7]. Disruption of these mechanically encoded, multiscale structures give rise to aberrant epithelial morphogenesis, which is increasingly recognized as an early structural precursor to epithelial developmental process or pathophysiology [8], [9]. Accordingly, deciphering how wrinkle structures and nuclear morphology jointly evolve during morphogenesis is critical for understanding the structural origins of epithelial development.

Despite its central role in epithelial development and pathophysiology, epithelial morphogenesis remains difficult to identify and characterize using conventional experimental models, which are predominantly based on in vivo animal systems, due to limited experimental controllability. Moreover, visualization of epithelial wrinkling and morphogenetic breakdown in vivo is further hindered by tissue motion, restricted optical access, and the transient nature of folding events. Consequently, the onset and progression of epithelial morphogenesis, as well as its coupling to tissue-scale mechanics and subcellular nuclear morphology, remain poorly understood.

Recently developed in vitro epithelial wrinkle tissues, engineered through morphomechanical approaches that recapitulate physiological contractile forces, have emerged as a promising platform to overcome the limitations of conventional in vivo models. Unlike in vivo systems, these platforms enable diverse in vitro analyses across morphological, genetic, and proteomic levels, while allowing precise control over experimental conditions, thereby facilitating systematic investigation of epithelial morphogenesis. Moreover, integration of engineered wrinkle structures with human-derived organoids provides physiologically relevant systems for controlled induction of morphogenesis and its dysregulation [10], [11], [12], [13]. Despite this promise, their broader application and scalability remain fundamentally constrained by the lack of scalable, label-free imaging strategies. Current approaches rely heavily on fluorescence microscopy [14], [15], [16], which necessitates exogenous labelling and labor-intensive sample preparation that can perturb native tissue states, hinder longitudinal studies, and compromise quantitative reproducibility. Furthermore, although fluorescence microscopy enables nuclear contrast and depth-resolved imaging via z-stack acquisition, its reliance on repeated optical sectioning and complex workflows severely limits throughput and scalability, constituting a major bottleneck for systematic multiscale analysis.

Label-free imaging modalities offer a compelling alternative for probing epithelial tissue structures, circumventing the complex and labor-intensive procedures required for conventional immunofluorescence staining. Photoacoustic microscopy (PAM), a representative label-free technique, converts optical absorption into ultrasonic signals, enabling optical contrast with ultrasonic depth resolution [17], [18], [19], [20], [21], [22], [23], [24]. In particular, ultraviolet photoacoustic microscopy (UV-PAM) employs 266-nm pulsed excitation that coincides with the strong intrinsic absorption of nucleic acids, enabling high-contrast visualization of cell nuclei without staining or physical sectioning [25], [26], [27], [28], [29], [30]. Recent advances have demonstrated that UV-PAM can achieve subcellular-resolution, label-free imaging of nuclear morphology in intact biological tissues, supporting histology-like visualization and quantitative analysis at the single-cell level [25], [26]. This unique combination of intrinsic nuclear contrast, volumetric sectioning, and compatibility with tissues positions UV-PAM as a compelling candidate for structural interrogation of aberrant epithelial morphogenesis. Nevertheless, its application to engineered in vitro epithelial systems for resolving multiscale structures remains largely unexplored, and has not yet been demonstrated in tissues with complex morphologies such as wrinkles or in organoid-based epithelial models.

Here, we introduce UV-PAM as a label-free, high-resolution imaging framework for multiscale structural analysis of organoid-based in vitro epithelial systems. This approach enables simultaneous, depth-resolved visualization of tissue-scale wrinkle structures and subcellular nuclear deformation in tissue samples. Moreover, using a controlled perturbation model of epithelial disruption, we demonstrate that UV-PAM sensitively captures early structural degradation in wrinkled epithelial organoid systems, as exemplified in intestinal organoids, prior to overt loss of tissue integrity [31], [32]. To enable automated and scalable phenotyping of epithelial morphogenesis, we further integrate UV-PAM with deep learning–based classification [33], [34], [35], [36], supporting preliminary discrimination between intact and chemically perturbed epithelial tissues using intrinsic, label-free structural features. This analysis was designed as a downstream computational assessment of whether UV-PAM images contain separable structural features associated with wrinkle-intact and chemically perturbed epithelial states. Grad-CAM + + and occlusion-sensitivity analyses were used to identify localized image regions contributing to the model predictions [37], [38]. Collectively, these results highlight the potential of UV-PAM combined with deep learning as a structure-based, label-free framework for imaging and computational phenotyping of epithelial morphogenesis.

## 2 Results

### 2.1 Morphomechanically defined 3D epithelial wrinkle tissue as in vitro baseline

**Fig. 1:**
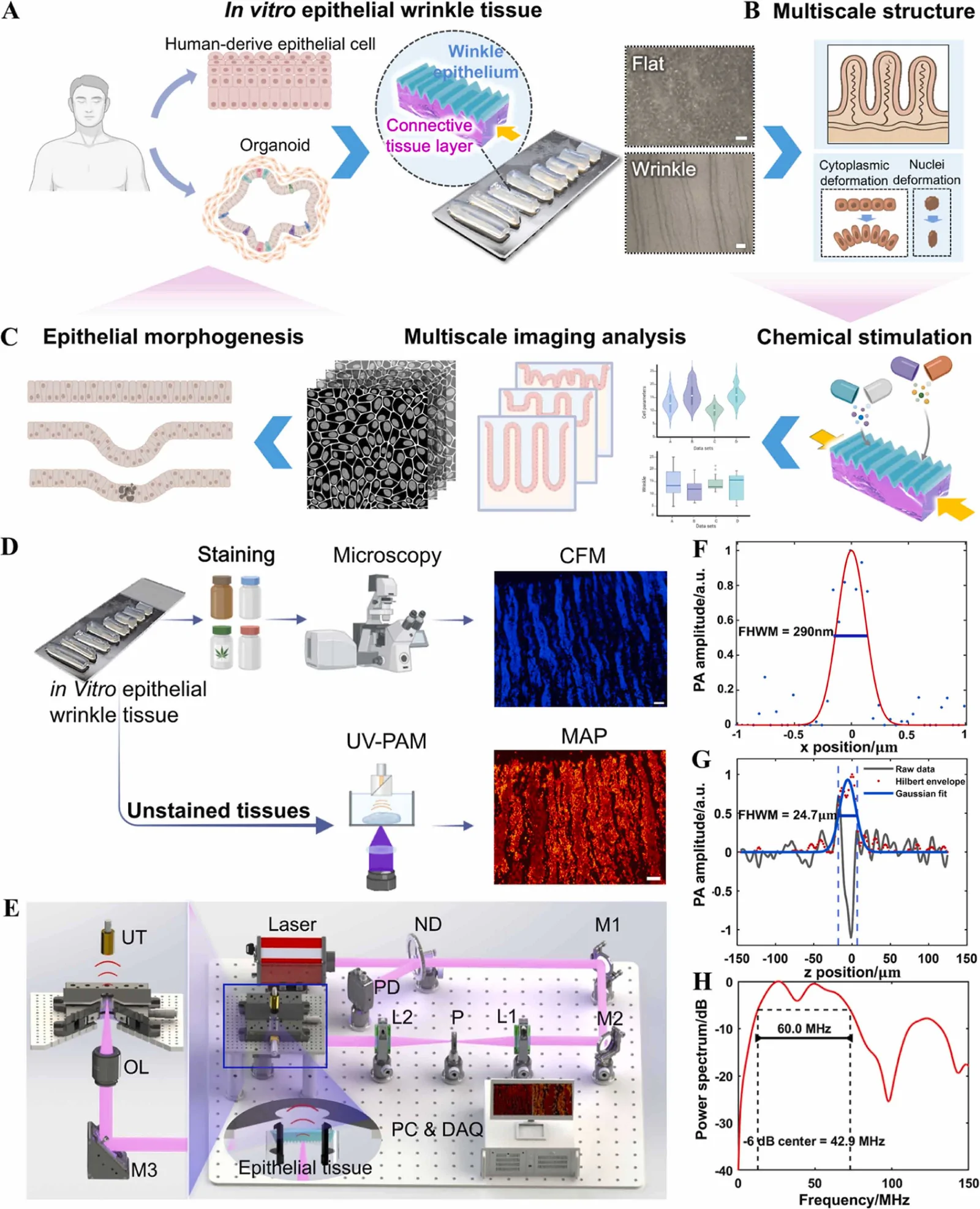
Schematic overview of label-free UV-PAM of 3D epithelial wrinkle tissues in vitro. A. Schematic illustration of engineered epithelial wrinkle tissues in vitro. Human-derived or organoid-derived epithelial cells are cultured to form epithelial–ECM bilayers, which undergo controlled compressive strain to generate epithelial wrinkle structures on a connective tissue layer. Representative flat and wrinkle morphologies of the engineered tissues are shown on the right. Scale bar, 100 µm. B. Schematic diagram of tissue-scale and subcellular-scale structure of epithelial wrinkle tissue under compression. C. Illustration of multiscale imaging of epithelial wrinkle tissues under chemical stimulation. Quantitative analysis enables characterization of structural changes associated with epithelial morphogenesis. D. Comparison of conventional fluorescence microscopy and label-free UV-PAM for imaging in vitro epithelial wrinkle tissues. CFM, Confocal microscopy. MAP, maximum amplitude projection. Scale bar, 100 µm. E. Experimental setup of the UV-PAM system. ND, neutral-density filter. M1-M3, mirrors. L1 and L2, Lenses. P, pinhole. PD, photodiode. OL, objective lens. UT, ultrasound transducer. PC & DAQ, personal computer and digital acquisition system. F. Lateral resolution measurement (FWHM = 290 nm) using a 100 nm gold nanoparticle. G. Axial resolution (FWHM = 24.7 µm) using a carbon fiber suspended approximately 2 mm above the substrate. It presents raw photoacoustic A-line signal, Hilbert envelope, and Gaussian fit results. H. Frequency spectrum of the photoacoustic signal generated from G.

To establish a morphomechanically defined in vitro platform that recapitulates compressive force-mediated epithelial wrinkle morphogenesis, we fabricated two types of engineered in vitro epithelial wrinkle tissues based on our previous work [39]. Specifically, epithelial tissues were constructed according to the cell source using either human-derived lung epithelial A549 cells or organoid-derived intestinal epithelial cells. These epithelial layers were integrated with a stimuli-responsive, contractile poly(N-isopropylacrylamide) (pNIPAAm) hydrogel layer that mimics smooth muscle-like contraction, thereby generating controllable compressive strain around 0.5 and enabling reproducible modulation of epithelial structure from flat monolayers to three-dimensional (3D) wrinkled morphologies (Fig. 1A). Detailed fabrication procedures are provided in the Methods.

These engineered epithelial wrinkle tissues recapitulate key multiscale structural features of mechanically deformed epithelial systems. At the tissue scale, compressive strain induces wrinkle structures in the epithelial layer, whereas at the subcellular scale it is associated with cytoplasmic and nuclear deformation (Fig. 1B). Such multiscale structural remodelling is highly relevant to epithelial development, homeostasis, and mechanically regulated cellular behavior. In native epithelial tissues of the gastrointestinal tract, including the stomach, small intestine, and colon, similar mechanical forces generated by underlying smooth muscle can be transmitted to the epithelium and connective tissue, driving strain-induced structural deformation across multiple length scales. This morphomechanical wrinkling is essential for epithelial morphogenesis, including airway and intestinal villus formation, and may also influence mechanotransductive signalling through nuclear deformation. Based on this engineered platform and its multiscale structural organization, we used these epithelial wrinkle tissues as a controllable model for investigating epithelial morphogenesis, performing multiscale imaging analysis, and probing chemically induced structural perturbations (Fig. 1C). These morphomechanically defined epithelial wrinkle tissues therefore served as a structural baseline for subsequent UV-PAM imaging and quantitative analysis.

### 2.2 Label-free UV-PAM of 3D in vitro epithelial wrinkle tissue

To enable label-free, depth-resolved interrogation of epithelial wrinkle tissues, we employed UV-PAM, which exploits the strong intrinsic absorption of nucleic acids at 266 nm (Fig. 1D). UV-PAM provides intrinsic nuclear contrast and 3D structural information directly from photoacoustic signals, eliminating the need for exogenous fluorescence labeling and iterative optical sectioning typically required for 3D reconstruction of wrinkle structures using conventional microscopy. A 266-nm nanosecond pulsed laser was delivered through a 4 f optical system and tightly focused onto the epithelial wrinkle tissue, and the generated photoacoustic signals were detected by a focused ultrasound transducer (Fig. 1E). Spatial lateral resolution was experimentally calibrated using 100-nm gold nanoparticles (Supplementary Fig. S1). The point spread function was quantified by full width at half maximum (FWHM) analysis (Fig. 1F), yielding a lateral resolution of ~290 nm, sufficient to clearly resolve wrinkle structures and individual cell nuclei within epithelial tissues. The axial resolution was evaluated using a suspended carbon-fiber target, resulting in an axial FWHM of 24.7 µm and an effective bandwidth of 60 MHz (Fig. 1G-H; Supplementary Fig. S1). This value is in reasonable agreement with the theoretical axial resolution of approximately 22 µm estimated from the measured bandwidth.

### 2.3 Benchmarking UV-PAM against confocal microscopy in human-derived lung epithelial wrinkle tissues

**Fig. 2:**
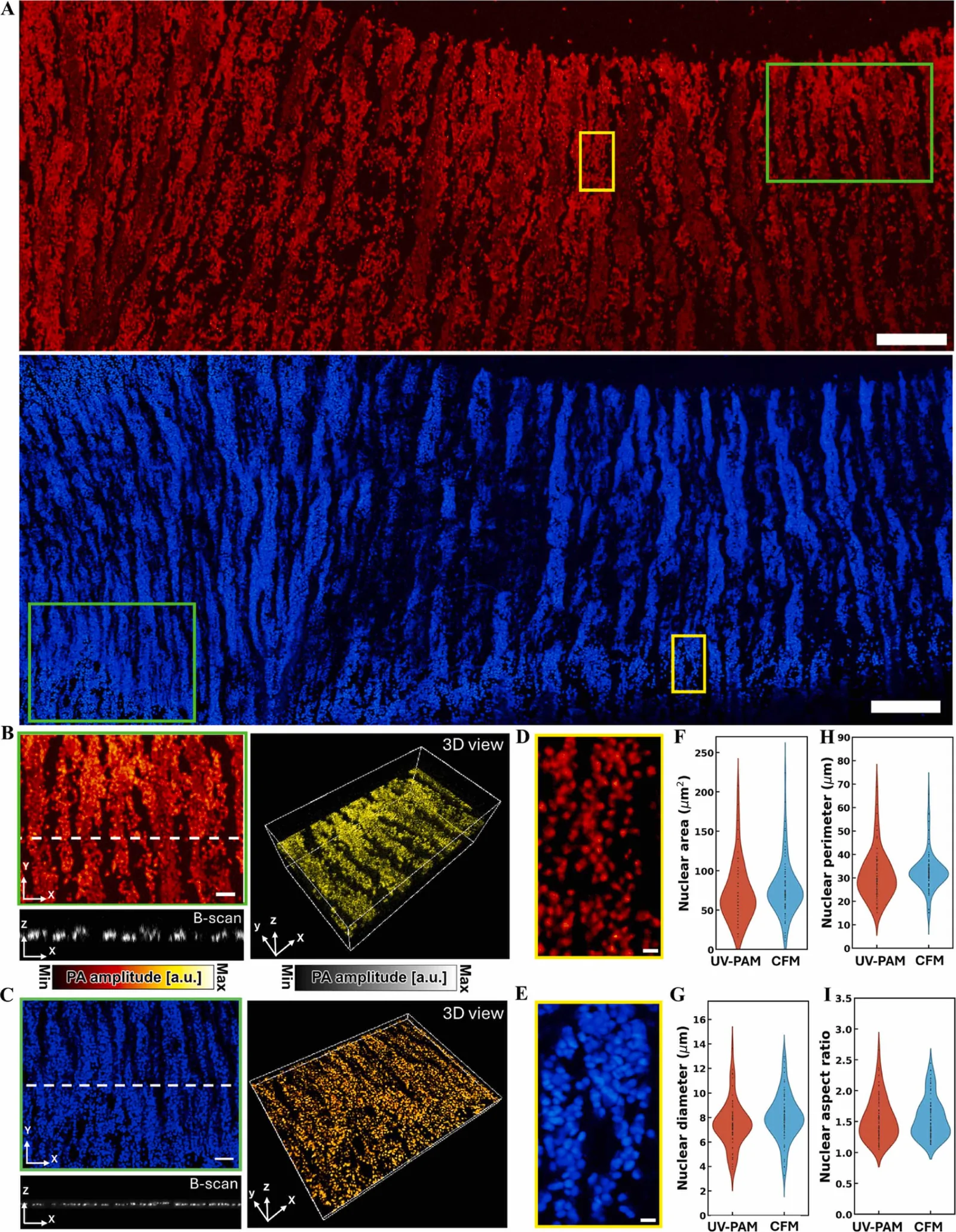
Imaging capability of UV-PAM compared with the confocal microscopy (CFM) in human-derived lung epithelial wrinkle tissue. A. Representative large FOV images of epithelial wrinkle tissues acquired by UV-PAM (top, unstained) and CFM (bottom, after DAPI staining). Scale bar, 300 µm. B. Enlarged MAP, B-scan profile, and 3D view obtained by UV-PAM from the large green box in A, acquired with a reduced step size of 1.25 µm. B-scan profiles are extracted along the white dashed line. Scale bar, 60 µm. C. Corresponding enlarged 2D image, B-scan profile, and 3D view acquired by CFM from the large green box in A. Scale bar, 60 µm. D. High-resolution MAP acquired from the small yellow box in A with a further smaller step size of 0.625 µm. E. Corresponding CFM images are cropped from the small yellow box in A. Scale bar, 15 µm. F–I. Quantitative analysis of nuclei area (F), diameter (G), perimeter (H), and aspect ratio (I) by UV-PAM and CFM derived from D and E.

To benchmark UV-PAM against DAPI(4′,6-diamidino-2-phenylindole)-based confocal fluorescence microscopy (CFM) [40], [41], both modalities were applied to the same epithelial wrinkle tissue. UV-PAM was performed first on the unstained specimen, after which the human-derived lung epithelial wrinkle tissue was DAPI-stained for CFM. During this process, staining, washing, and remounting procedures between the two imaging steps can introduce slight positional shifts of the tissue relative to the imaging stage, making precise relocation of the identical imaging region challenging. Therefore, representative regions exhibiting comparable wrinkle structures were selected for comparison. Both modalities resolve a similar periodic ridge–valley organization of epithelial wrinkles, with comparable wrinkle spacing maintained across a large field of view (FOV), indicating tissue-scale uniformity. In addition, the wrinkle structures exhibit pronounced anisotropic alignment orthogonal to compression rather than random orientation, reflecting organized mechanical patterning at the tissue level. To examine structural features across spatial scales, two representative subregions highlighted by the large green rectangles in the top (UV-PAM) and bottom (CFM) panels of Fig. 2A were selected, with the corresponding UV-PAM MAP (maximum amplitude projection) and 3D view shown in Fig. 2B (left and right, respectively) and the matched CFM results presented in Fig. 2C in the same layout, enabling clearer visualization of wrinkle morphology. The 3D view provides a more intuitive visualization of the ridge–valley topology and subtle surface undulations of wrinkle structures. B-scan profiles of UV-PAM and CFM results in Fig. 2B and 2C were extracted along the white dashed lines, revealing height modulations corresponding to wrinkle amplitude.

To further investigate nuclear morphology within wrinkle tissues, regions marked by the small yellow boxes in the top (UV-PAM) and bottom (CFM) panels of Fig. 2A were selected for higher-resolution analysis. The corresponding UV-PAM and CFM images clearly resolve epithelial nuclei aligned along the wrinkle structures, particularly showing orientation parallel to the wrinkle pattern (Fig. 2D and 2E). UV-PAM visualizes nuclei through intrinsic nucleic-acid absorption without labelling, whereas CFM visualizes nuclei through DAPI fluorescence staining. Within the analyzed tissue specimen, UV-PAM and CFM yielded broadly comparable distributions of nuclear area, diameter, perimeter, and aspect ratio, supporting the feasibility of UV-PAM for label-free nuclear morphological characterization (Fig. 2F–I and Supplementary Table 1). Representative UV-PAM images obtained from an additional living epithelial wrinkle tissue are provided in Supplementary Fig. S2, demonstrating the capability of UV-PAM to visualize nuclear morphology within live wrinkle tissues. These results demonstrate that UV-PAM provides high-resolution, label-free visualization of anisotropically aligned, approximately periodic wrinkle structures and nuclear layers, supporting its utility for multiscale structural analysis without the need for exogenous staining.

### 2.4 Mechanical compression couples tissue-scale wrinkling with nuclear remodelling

**Fig. 3:**
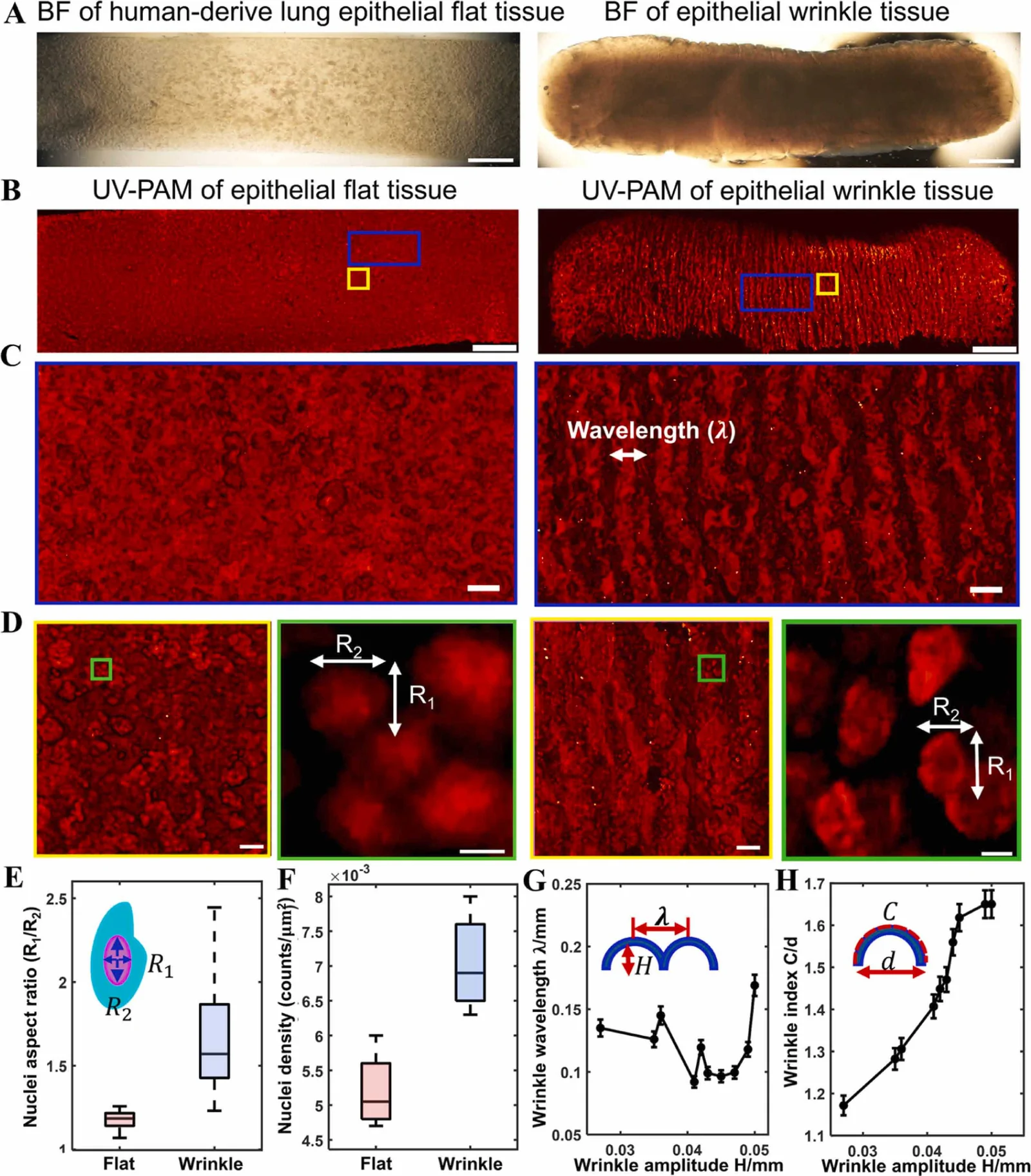
UV-PAM reveals multiscale structural remodeling of human-derived lung epithelial wrinkle tissues under compressive strain. A. Bright-field microscopy (BFM) of wrinkle tissues without (left, flat)/ with (right, wrinkle) compressive strains. Scale bar, 1 mm. B. Large FOV MAPs of epithelial flat and wrinkle tissues. Scale bar, 1 mm. C. Enlarged MAPs corresponding to the blue rectangles in flat and wrinkle tissues in B, acquired with a reduced step size of 1.25 µm. Scale bar, 100 µm. D. D1 and D3. High-resolution MAPs acquired from the yellow square regions of flat and wrinkle tissues in B with a smaller step size of 0.625 µm. D2 and D4. Magnified views extracted from the green boxes in D1 and D3. The magnified views highlight changes in cell shape following compressive strain. Scale bar: 50 µm (high-resolution MAPs); 5 µm (close-ups). E–F. Quantitative analysis of nuclear aspect ratio (E) and nuclear density (F) of epithelial flat and wrinkle tissue in D. G. Relationship between wrinkle wavelength and wrinkle amplitude quantified from the enlarged MAPs in C. H. Wrinkle index-wrinkle amplitude curve of epithelial wrinkle tissues in C. Wrinkle index is calculated by the ratio between the perimeter and width of each wrinkle structure. The error bars in Fig. 3G–H represent the standard deviation across three within-tissue technical measurements for each wrinkle parameter.

To quantitatively assess how compression modulates epithelial wrinkle structure across scales, we compared human-derived lung epithelial tissues cultured under two defined conditions: a flat epithelial tissue without mechanical compression and a morphomechanically compressed epithelial tissue exhibiting wrinkle structure. Both models were imaged using UV-PAM to determine whether tissue-scale wrinkling and associated subcellular nuclear deformation could be resolved simultaneously within the same imaging framework. Bright-field images provide an overall view of the epithelial tissues, whereas the corresponding UV-PAM MAPs clearly resolve wrinkle structures across the epithelial layer at the tissue scale (Fig. 3A and 3B). In contrast to the largely flat morphology of uncompressed epithelium, compressive strain produces well-defined periodic wrinkle patterns. To highlight the structural difference within the two tissues, two representative subregions highlighted by the blue rectangles in left (flat) and right panels (wrinkle) of Fig. 3B were selected for higher-resolution analysis. The corresponding enlarged MAPs provide clearer visualization of characteristic flat and wrinkle-rich morphologies (Fig. 3C). Depth-encoded MAPs generated from the corresponding volumetric datasets further visualized the relative depth variations across the flat and compressed tissues (Supplementary Fig. S3). The flat tissue showed a relatively irregular depth distribution without a clear periodic pattern (Supplementary Fig. S3A), whereas the mechanically compressed tissue exhibited spatially organized depth variations associated with the ridge–valley morphology of the wrinkle structures (Supplementary Fig. S3B). These depth-encoded maps provide complementary visualization of the topographic differences between the flat and compressed tissues. To interrogate nuclear morphology, the regions indicated by yellow squares in Fig. 3B were analysed, with the corresponding high-resolution MAPs in Fig. 3D1 and 3D3. Magnified views extracted from the green squares elucidate direct visualization of nuclear morphology under uncompressed and compressed strain (Figs. 3D2 and 3D4). Compared with nuclei in flat tissues, nuclei in wrinkle tissues appear more elongated and more densely distributed, indicating strain-induced nuclear deformation.

Quantitative analysis confirms the visualized observations, the mean nuclear aspect ratio increases from around 1.23 in flat tissues to 1.68 in wrinkle tissues, and the mean nuclear density increases from about 5.2 × 10³ to 7 × 10³ counts mm⁻², indicating strain-induced nuclear elongation and denser nuclear distribution (Fig. 3E and 3F). As a complementary descriptive summary, the median nuclear aspect ratio increased from 1.24 to 1.57, and the median nuclear density increased from 5.05 × 10^3^–6.90 × 10^3^ counts mm^−2^, corresponding to relative differences of 26.22% and 36.63%, respectively (Supplementary Table 2). Beyond subcellular metrics, UV-PAM further enables quantitative characterization of tissue-scale wrinkle organization. Wrinkle wavelength,

wrinkle index, and wrinkle amplitude are quantified from the enlarged MAPs in Fig. 3C. The wrinkle wavelengths ranged from approximately 100 µm to 180 µm, with corresponding amplitudes ranging from approximately 26 µm to 51 µm, revealing the range of tissue-scale deformation observed under compression (Fig. 3G). Meanwhile, the wrinkle index increases from around 1.18–1.65 with increasing wrinkle amplitude, capturing increasing morphological complexity of the wrinkle structure. (Fig. 3H). Wrinkle amplitude, reflecting the peak-to-valley height modulation of the wrinkle structure, was extracted from UV-PAM B-scan profiles (Supplementary Fig. S4). These results demonstrate that UV-PAM simultaneously resolves subcellular nuclear morphology and tissue-scale wrinkle structure, enabling multiscale, quantitative structural analysis of epithelial wrinkle tissues under defined mechanical strain.

### 2.5 UV-PAM reveals structural signatures of dysregulated epithelial morphogenesis

**Fig. 4:**
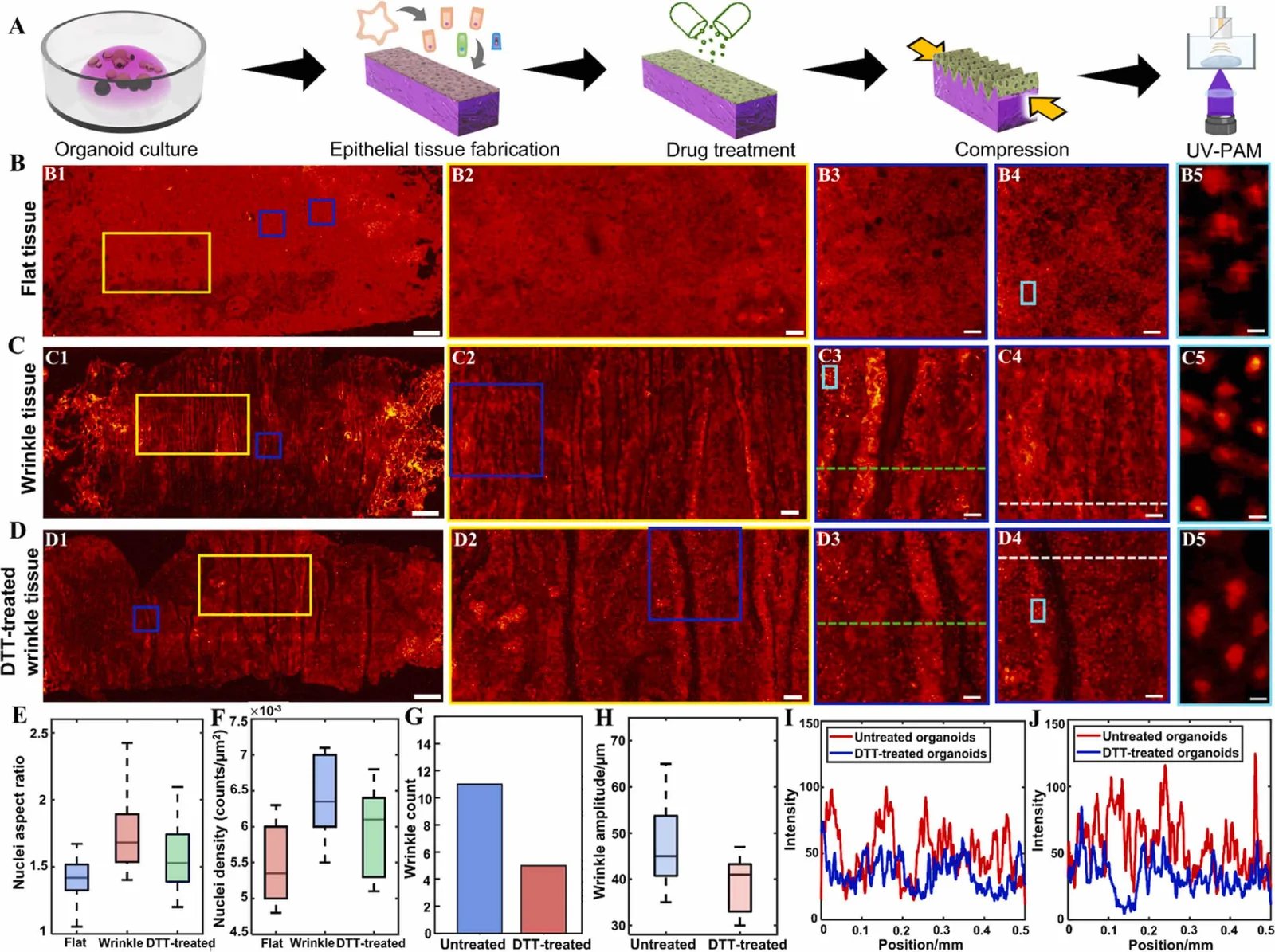
UV-PAM captures chemical-induced degradation of wrinkle structures in organoid-derived intestinal epithelial wrinkle tissue. A. Schematic illustration of organoid-derived intestinal epithelial wrinkle tissue generation, morphomechanical compression, and dithiothreitol (DTT) treatment. B. Multiscale MAPs of flat tissue without compressive strain. B1. Large FOV MAPs of the flat tissue. B2-B4. Enlarged MAPs extracted from the yellow rectangle and blue squares in B1. B5. Cyan-boxed close-up view extracted from B4. Scale bar, 500 µm (B1), 100 µm (B2), 50 µm (B3 and B4), 10 µm (B5). C. Multiscale MAPs of wrinkle tissues with compressive strain. C1. Large FOV MAPs of the wrinkle tissue. C2-C4. Enlarged MAPs extracted from the yellow rectangle and blue squares in C1 and C2. C5. Cyan-boxed close-up view extracted from C3. Scale bar, 500 µm (C1), 100 µm (C2), 50 µm (C3 and C4), 10 µm (C5). D. Multiscale MAPs of wrinkle tissue after DTT treatment. D1. Large FOV MAPs of DTT-treated wrinkle tissue. D2-D4. Enlarged MAPs extracted from the yellow rectangle and blue squares in D1 and D2. D5. Cyan-boxed close-up view extracted from D4. Scale bar, 500 µm (D1), 100 µm (D2), 50 µm (D3 and D4), 10 µm (D5). E-F. Quantitative analysis of nuclei aspect ratio (E) and density (F) derived from high-resolution MAPs in B, C and D. G–H. Wrinkle counts (G) and wrinkle amplitude (H) of untreated and DTT-treated tissues, calculated from enlarged MAPs in C and D. I–J. Intensity profiles were extracted from line scans along the dashed lines in C and D. Profiles in I correspond to the green dashed lines in C3 and D3, whereas profiles in **J** correspond to the white dashed lines in C4 and D4, and were smoothed for visualization.

To assess the capability of UV-PAM to detect pathological remodelling of epithelial structure, we employed a controlled chemical perturbation model in organoid-derived intestinal epithelial wrinkle tissues. Dithiothreitol (DTT), a reducing agent that disrupts protein disulfide bonds, was used to perturb epithelial integrity and induce structural and cytoskeletal alterations [10]. As illustrated schematically in Fig. 4A, organoid-derived epithelial bilayers initially exhibited a flat morphology prior to mechanical stimulation. Upon application of compressive strain, the bilayers developed pronounced wrinkle structures, both in the absence and presence of DTT treatment. UV-PAM MAPs reveal three distinct structural states: flat epithelial bilayers without compression, wrinkled bilayers under compressive strain, and wrinkled bilayers following DTT treatment (Fig. 4B1-D1). Compared with untreated organoid-derived intestinal epithelial wrinkle tissue, DTT-treated tissues exhibit markedly disrupted and flattened wrinkle structures, indicating chemical degradation of mechanically encoded structures.

To examine the structural changes quantitatively, representative subregions highlighted by the yellow rectangles in Fig. 4B1–D1 were selected for higher-resolution analysis (Fig. 4B2–D2). The corresponding MAPs reveal clear differences in wrinkle morphology between flat tissues, untreated wrinkle tissues, and DTT-treated wrinkle tissues. Untreated tissues exhibit well-defined wrinkle ridges and valleys, whereas DTT-treated tissues display locally flattened and disrupted wrinkle structures. To further resolve subcellular features, high-resolution MAPs were extracted from the blue-boxed regions in Fig. 4B–D, with corresponding magnified views in Fig. 4B3–D4. The cyan-marked magnified areas in Fig. 4B5–D5 clearly reveal epithelial nuclei across flat tissues, untreated wrinkle tissues, and DTT-treated wrinkle tissues. Compared with flat tissues, nuclei in untreated wrinkle tissues appear more elongated and aligned along wrinkle ridges, whereas DTT-treated tissues exhibit partially disrupted wrinkle patterns and dispersed nuclear distributions. In addition, Supplementary Fig. S5 provides the corresponding CFM images of untreated and DTT-treated tissues as an independent reference to validate nuclear morphological differences observed by UV-PAM.

Quantitative analysis demonstrated that compressive strain induces increases in mean nuclear aspect ratio from approximately 1.4–1.8 and mean nuclear density from around 5.5 × 10³ to 6.4 × 10³ counts mm⁻² relative to flat tissues, indicating the strain-driven nuclear elongation and compaction (Fig. 4E and 4F). The DTT- treated group

exhibited lower median nuclear aspect ratio and nuclear density than the untreated wrinkle group, while both values remained above those of the flat group. DTT treatment further leads to reductions in both wrinkle count and wrinkle amplitude, with wrinkle count decreasing from approximately 11–5 and mean wrinkle amplitude decreasing from around 48 µm to 39 µm, reflecting disruption of mechanically encoded wrinkle structures (Fig. 4G and 4H). Complementary median analysis showed reductions of 9.02% in nuclear aspect ratio and 3.94% in nuclear density in DTT-treated tissues relative to untreated wrinkle tissues (Supplementary Table 3). Consistent with the structural changes, intensity profiles were extracted from line scans across wrinkle regions in Fig. 4C and 4D. Profiles were extracted along representative wrinkle ridges and valleys to capture characteristic peak–valley intensity variations of the wrinkle structure. Profiles in Fig. 4I correspond to the green dashed lines in Fig. 4C3 and 4D3, whereas profiles in Fig. 4J are obtained from the white dashed lines in Fig. 4C4 and 4D4. The resulting profiles showed a lower overall signal level and fewer peaks in DTT-treated tissues. Because the traces also differed in global amplitude, these profiles were interpreted descriptively together with the corresponding MAP-based structural measurements (Fig. 4I and 4J). Collectively, these results demonstrate that UV-PAM sensitively captures chemical induced degradation of mechanically encoded wrinkle structures in organoid-derived intestinal epithelial wrinkle tissues, revealing multiscale structural signatures of dysregulated epithelial morphogenesis in a label-free manner.

### 2.6 AI-ready structural phenotyping of epithelial wrinkle tissues enabled by UV-PAM

**Fig. 5:**
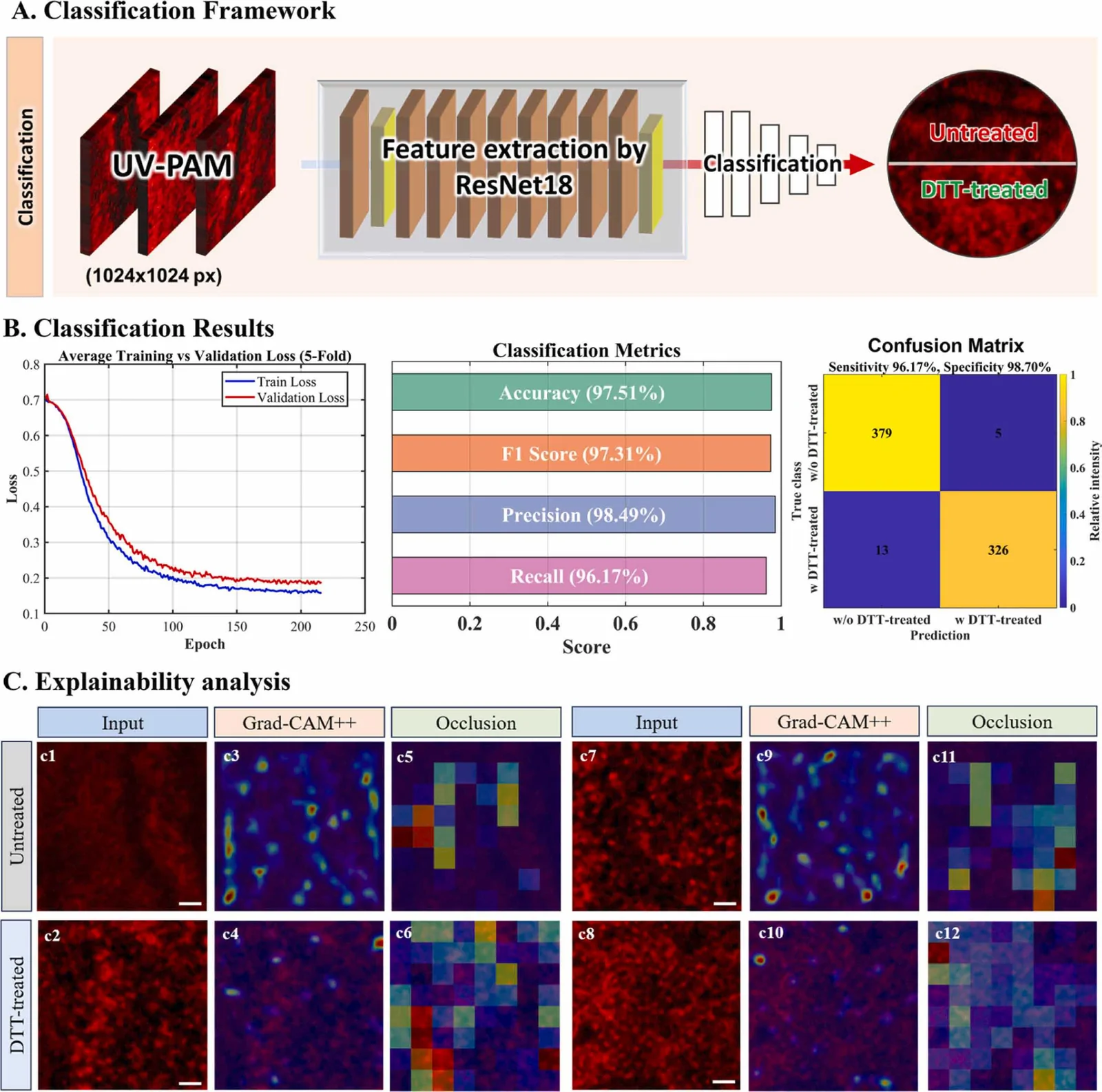
Deep learning-based classification of organoid-derived intestinal epithelial wrinkle tissues with/without DTT treatment. A. Schematic overview of the ResNet18 framework trained on UV-PAM images (1024 × 1024 pixels) to discriminate untreated from DTT-treated wrinkle tissues. B. Training and classification performance. Average training and validation loss curves (left) indicate stable convergence under 5-fold cross-validation. Quantitative metrics (center) show high accuracy (97.51%), F1 score (97.31%). The confusion matrix (right) shows high patch-level sensitivity (96.17%) and specificity (98.70%) with few misclassifications. C. Grad-CAM++ and occlusion-sensitivity analysis; representative untreated and DTT-treated UV-PAM patches are shown with corresponding Grad-CAM++ and occlusion maps. Warmer colors indicate regions with greater influence on the predicted class. Scale bars, 100 μm.

To determine whether the intrinsic, label-free structural signatures resolved by UV-PAM contain discriminative information suitable for automated analysis, we integrated UV-PAM imaging with a deep learning–based classification framework. Rather than treating deep learning purely as a classification tool, this analysis was designed as a preliminary assessment of whether UV-PAM images contain discriminative structural patterns beyond those captured by individual predefined morphological parameters, thereby supporting the feasibility of computational phenotyping within the available specimens. Specifically, we employed a ResNet18–based [42] convolutional neural network to classify UV-PAM images of untreated and DTT-treated tissues, corresponding to wrinkle-intact and wrinkle-compromised epithelial states. The dataset comprised two untreated and two DTT-treated specimens. Before image tiling, five FOVs per class were assigned to the training set and three FOVs per class to the fixed test set. The training and test FOVs were obtained from the same specimens. The FOVs were subsequently divided into 1024 × 1024-pixel patches, followed by fine-tuning of the ResNet18 backbone (Fig. 5A). Through hierarchical feature extraction, the network learned multiscale image patterns that supported patch-level discrimination between untreated and DTT-treated conditions on held-out FOVs. Because the 723 test patches were generated with 50% overlap from held-out FOVs of the same specimens, they were not considered independent biological observations.

During training, both training and validation losses decreased steadily after approximately 20 epochs and subsequently reached stable plateaus, indicating stable optimization within the training and validation folds (Fig. 5B, left panel). The smooth decay and subsequent plateau of the validation loss across the five folds further support the stability of the optimization procedure. In testing, the model achieved high performance, with an accuracy of 97.51%, F1 score of 97.31%, precision of 98.49%, and recall of 96.17% (Fig. 5B, centre and right panel). The confusion matrix showed only a few misclassifications, corresponding to a sensitivity of 96.17% for detecting DTT-treated organoids and a specificity of 98.70% for identifying untreated tissues, showing high patch-level discrimination between the two conditions within the evaluated dataset. Among the 18 misclassified test patches, five untreated patches were predicted as DTT-treated and 13 DTT-treated patches as untreated (Supplementary Fig. S6). In both groups, the patches showed limited spatially coherent ridge–valley organization, resulting in overlapping local appearances between the two conditions. The false-negative examples had predictions close to the decision threshold (0.513 and 0.510; Fig. S6A), whereas the false-positive examples showed higher confidence (0.849 and 0.627; Fig. S6B). Corresponding Grad-CAM++ maps exhibited sparse, localized activations rather than responses spanning organized wrinkle regions, suggesting limited discriminative structure within these patches. The predominance of false negatives may reflect spatial heterogeneity in DTT-induced disruption, with some treated patches retaining locally intact wrinkle-like structures. These results represent proof-of-concept patch-level performance on held-out FOVs from the available specimens. To examine whether the classification performance could be explained by global image intensity, untreated and DTT-treated patches showed mean normalized red-channel intensities of 0.3457 ± 0.0870 and 0.3250 ± 0.0702 (Supplementary Fig. S7), respectively. A logistic-regression classifier using mean intensity alone achieved 52.15% accuracy, while a classifier using seven global intensity features achieved 58.64%, compared with 97.51% for the ResNet18 ensemble. These findings indicate that global brightness alone was insufficient to reproduce the deep-learning performance. Saturated pixels were defined as those with normalized red-channel intensity ≥ 254/255. Among 586 test patches containing saturated pixels, masking the saturated regions changed the predicted class in only 16 cases (2.7%), indicating that saturated signals alone did not generally determine the predictions (Supplementary Table 4).

To further investigate model decision-making, Grad-CAM++ and occlusion-sensitivity analyses were performed (Fig. 5C). Grad-CAM++ identified localized regions contributing to the predicted class, while occlusion of the highest-attribution regions produced a greater decrease in predicted-class confidence than occlusion of size-matched random regions (0.0273 ± 0.0289 versus 0.0063 ± 0.0139). These findings indicate that the highlighted regions contributed to model predictions, although they were not interpreted as exclusively representing complete wrinkle ridges. We additionally evaluated classification performance using CFM images (Supplementary Fig. S8) and compared the results with UV-PAM-based classification (Supplementary Table 5). Additional cross-validation, receiver operating characteristic, and precision–recall analyses further characterized classification performance within the evaluated dataset (Supplementary Fig. S9). These results support the feasibility of combining UV-PAM with deep learning for patch-level computational phenotyping of epithelial wrinkle states in the present setting.

## 3 Discussion

High-resolution, multiscale structural characterization of organoid-derived epithelial wrinkle tissues is essential for understanding how tissue mechanics, nuclear organization, and epithelial morphogenesis are coordinated in vivo [43]. In this work, we demonstrate that UV-PAM enables label-free, depth-resolved imaging of both tissue-scale wrinkle structures and subcellular nuclear morphology within mechanically and chemically regulated epithelial systems. By exploiting intrinsic ultraviolet absorption of nucleic acids, UV-PAM provides direct nuclear contrast and volumetric sectioning without reliance on exogenous labelling or multi-step staining, facilitating quantitative structural analysis across samples.

Our results reveal that compressive strain induces coordinated remodelling across length scales, manifested as enhanced wrinkle formation at the tissue level together with nuclear elongation and increased nuclear distribution at the single-cell level. These nuclear morphological changes reflect strain-driven cytoskeletal reorganization and nuclear mechanotransduction, processes central to epithelial development and tissue homeostasis [44]. Chemical perturbation using DTT further demonstrates that disruption of mechanically encoded epithelial wrinkle organization is accompanied by collapse of wrinkle structures and attenuation of nuclear deformation. Such structural alterations are consistent with DTT-mediated reduction of disulfide bonds in epithelial cells and the mucus layer, which weakens cell–cell junctions and compromises wrinkle organization [10]. These results support the emergence of a label-free structural phenotype associated with dysregulated epithelial morphogenesis.

Integration of UV-PAM with deep learning further demonstrates the potential computational utility of UV-PAM-derived structural images for automated phenotypic analysis. The ResNet18-based classifier provided preliminary evidence that intrinsic structural signatures resolved by UV-PAM support patch-level discrimination between wrinkle-intact and wrinkle-compromised epithelial states within the available specimens. Misclassified patches were confined to regions in which wrinkle organization was locally ambiguous between untreated and DTT-treated tissues, and the false-negative cases occurred at predicted probabilities close to the decision threshold, suggesting that the residual errors may reflect local morphological overlap between the two conditions. Extending this framework to larger biological cohorts with complete specimen-level separation between training and testing is the natural next step towards establishing generalizability to unseen tissues. Beyond binary patch classification, a structure-aware multiscale framework that jointly incorporates tissue-level wrinkle architecture and nuclear-level morphology, with quantitative structural measurements used as auxiliary supervisory information, may further improve the biological interpretability of computational phenotyping. ResNet18 was selected to balance computational efficiency and classification performance [45], [46].

Imaging throughput is currently limited by mechanical scanning, future incorporation of fast galvanometric scanning strategies and metalens-enabled volumetric photoacoustic imaging may further enhance performance [47], [48], [49], [50]. The present UV-PAM system employed a transmission-mode configuration, which was suitable for the current study because the specimens were oriented with the epithelial wrinkle surface toward the imaging side and the primary analysis focused on surface morphology and relative wrinkle amplitude. Nevertheless, for more thicker specimens, propagation of both the excitation light and generated acoustic waves through the sample may lead to depth-dependent attenuation and reduced signal-to-noise ratio. Reflection-mode UV-PAM configurations, in which optical excitation and acoustic detection are arranged on the same side of the specimen, can reduce these transmission-related constraints and are generally better suited for thicker or irregularly shaped samples [51], [52], [53], [54]. Future implementation of a reflection-mode geometry may therefore improve signal-to-noise ratio and enable more precise depth characterization of volumetric organoid and tissue samples. Repeated-imaging performance is another important consideration for future dynamic applications. Four consecutive scans of the same tissue region showed recognizable wrinkle and nuclear structures. Quantitative analysis of nuclear area, perimeter, diameter, and aspect ratio showed no statistically significant differences between the first and subsequent acquisitions using Welch’s two-sample *t*-tests (all p > 0.05; p = 0.074–0.941), supporting short-term morphological repeatability within the examined ROI under the tested imaging conditions (Supplementary Fig. S10). Further post-imaging viability and DNA-damage assessments will be needed to establish safe exposure conditions for longitudinal imaging or repeated drug-response monitoring. Subject to such validation, coupling with organoid-on-chip platforms or in vivo models may ultimately enable dynamic studies of epithelial remodelling under physiological and pathological conditions.

## 4 Conclusions

In summary, we pioneered the application of UV-PAM to achieve label-free and high-resolution imaging of 3D organoid-derived epithelial wrinkle tissues. UV-PAM enables simultaneous visualization of tissue-scale wrinkle structures and quantitative characterization of subcellular nuclear morphology under mechanically and chemically regulated conditions. Using compressive strain and DTT-induced perturbation as controlled models, we show that UV-PAM sensitively captures multiscale structural remodelling associated with dysregulated epithelial morphogenesis. By integrating deep learning–based analysis, we further demonstrate in a proof-of-concept setting that UV-PAM-derived structural images support patch-level separation of wrinkle-intact and wrinkle-compromised epithelial states based on intrinsic morphological features. Additional intensity and saturation controls indicated that observed patch-level classification performance relied on image features beyond global brightness and saturated regions. Nevertheless, given the limited number of biological specimens, acquisition-related confounding cannot be completely excluded. Validation using larger biological cohorts and specimen-level held-out testing will further establish the reproducibility and generalizability of this computational phenotyping approach. These findings establish UV-PAM as a versatile imaging tool for mechanobiology and disease modelling in 3D epithelial systems, with potential applications in organoid-based functional analysis and future drug-response studies.

## 5 Materials and methods

### 5.1 Preparation of 3D in vitro epithelial wrinkle tissues

Epithelial tissues were constructed as bilayer structures composed of an ECM hydrogel substrate and epithelial layers. To define the hydrogel geometry, polydimethylsiloxane (PDMS) (Sylgard 184; Dow Corning, USA) molds were prepared by mixing PDMS base and curing agent at a 10:1 wt ratio, followed by degassing and thermal curing. Rat-tail type I collagen (Corning, USA) was neutralized with 1 M sodium hydroxide (NaOH; Sigma-Aldrich, USA) and 10 × Dulbecco’s modified Eagle’s medium (DMEM; Gibco, USA) at a volume ratio of 1:0.025:0.1 and adjusted to an appropriate working concentration before being cast into PDMS molds. Hydrogels were polymerized under standard cell culture conditions prior to subsequent cell seeding. For lung airway epithelial tissues, human alveolar epithelial cells lung (A549; Korean Cell Line Bank, Republic of Korea) were cultured in Roswell Park Memorial Institute 1640 (RPMI 1640; Gibco, USA) supplemented with 10% fetal bovine serum (FBS; Gibco, USA) and 1% amphotericin B (Sigma-Aldrich, USA). Cells were enzymatically detached, seeded onto the collagen hydrogels at 1 × 10⁶ cells mL^−1^, and maintained until confluent monolayers were formed. For colon epithelial tissues, the human colon organoid line CO4 (3D Organoids Core, Baylor College of Medicine, USA) was expanded in Matrigel droplets using complete organoid culture medium was consisted of a 1:1 mixture of high Wnt medium (3D Organoids Core, Baylor College of Medicine, USA) and advanced Dulbecco's modified Eagle medium (DMEM)/F-12 (Gibco, USA), supplemented with 1% amphotericin B. For monolayer formation, organoids were dissociated into single cells through enzymatic treatment and mechanical pipetting, followed by centrifugation and resuspension in fresh medium. The resulting single-cell suspension was seeded onto collagen hydrogels, where organoid-derived epithelial layers were established within 2–3 days.

To fabricate wrinkle epithelial tissues, a thermo-responsive compression system based on poly(N-isopropylacrylamide) (pNIPAAm) hydrogels was employed. The pNIPAAm precursor solution was prepared by dissolving N-isopropylacrylamide (NIPAAm, 97%; Sigma-Aldrich, USA) in DI water at a weight ratio 9:1, supplemented with 2 wt% N, N′-methylenebisacrylamide (MBAAm; Sigma-Aldrich, USA) and 0.05 wt% 2-hydroxy-1-[4-(2-hydroxyethoxy) phenyl]-2-methyl-1-propanone (Irgacure 2959; BASF, Germany). The solution was homogenized and cast into PDMS molds. Following UV irradiation (365 nm), polymerized hydrogels were demolded, shaped into circular discs using a laser cutting machine (IS350, INNOSTA, Republic of Korea), and equilibrated in aqueous solution and culture medium (Advanced DMEM; Gibco, USA) for 10 h at room temperature (RT) to induce swelling prior to biological use. Epithelial tissues were positioned onto the center of swollen pNIPAAm discs and allowed to adhere briefly before medium addition. Culture medium was then introduced to fully submerge the constructs, were incubated at 37 °C for 2 h to trigger thermally induced contraction of the pNIPAAm and generate wrinkle formation within the epithelial layers. For the preparation of 1,4-Dithiothreitol (DTT, Roche, Switzerland)-treated wrinkled epithelial tissues, epithelial constructs were preincubated in culture medium containing DTT for 1 h before compression. Specifically, cells were preincubated with 20 mM DTT (Roche Diagnostics, Switzerland) dissolved directly in the culture medium without an additional vehicle, for 1 h before compression. Control cells received the same culture medium without DTT.

### 5.2 Ultraviolet photoacoustic microscopy system

In the UV-PAM system, we employed a 266 nm pulsed laser (DX-266–0.05, Photonics Industries, USA) operating at 20 kHz with a maximum power of 50 mW. Residual 532 nm light was removed using a bandpass filter (FGUV5, Thorlabs, USA), and the beam intensity was controlled via a neutral-density filter (NDC-50C-4M, Thorlabs, USA). The average power measured at the sample plane was approximately 0.5 mW at a pulse repetition rate of 20 kHz, corresponding to a pulse energy of approximately 25 nJ. Using the measured lateral FWHM of 290 nm as an estimate of the excitation-beam FWHM and assuming a Gaussian excitation profile, the fluence average over Gaussian 1/e2 beam area was estimated to be approximately 13.1 J/cm2 per pulse. One laser pulse was used per pixel during raster scanning. At the minimum biological imaging step size of 0.625 µm, the estimated contribution from an adjacent Gaussian excitation spot was approximately 2.6 × 10^−6^ of the on-axis value, indicating negligible overlap between neighboring scan positions. Therefore, the local cumulative fluence during a single scan was approximately equal to the per-pulse fluence. A 10 μm pinhole (84–909, Edmund Optics, USA) together with planoconvex lenses (LA4052-UV and LA4102-UV, Thorlabs, USA) shaped and collimated the beam, while an iris diaphragm (SM1D25, Thorlabs, USA) suppressed peripheral diffraction. Two UV-enhanced mirrors (PF20–03-F01, Thorlabs, USA) directed the beam to an aspherical objective (AFL25–17-P-X, Asphericon, Germany, NA 0.64 at 355 nm) mounted on a motorized z-stage (CT1P/M, Thorlabs, USA) with 0.5 μm step resolution. Photoacoustic signals were collected by a single-element transducer (HFM23, Sonaxis, France, 50 MHz, 3 mm focal length, 90% bandwidth), amplified (DPR500, JSR Ultrasonics, USA), and filtered (3–150 MHz). Biological samples were stabilized in a custom aluminum holder with 0.2 mm quartz glass. Sample scanning was achieved via two-axis motorized stages (8MTF-75L-S05, Standa, Lithuania), controlled through LabVIEW 2021 (National Instruments, USA), with synchronized signal acquisition by a digitizer (ATS9352, Alazartech, Canada) and a multichannel I/O device (NI-PCIe-6361, National Instruments, USA).

### 5.3 Photoacoustic data acquisition and signal processing

Scanning was conducted in a raster pattern, acquiring B-line data along the x-axis while translating the sample along the y-axis to complete the 2D scan. The lateral scanning step size was adjustable and set to 5 μm, 1.25 μm, or 0.625 μm depending on the imaging requirement. The experimentally measured lateral FWHM of 290 nm represents the system point-spread function obtained under densely sampled phantom conditions. In contrast, all biological images were acquired with a minimum sampling interval of 0.625 μm. The collected binary datasets were post-processed in MATLAB R2024a (MathWorks, USA). Each A-line was bandpass filtered to remove high- and low-frequency noise, followed by Hilbert-transform envelope detection to reconstruct the 3D volume. MAP images were then generated, and a 2D median filter was applied to enhance image clarity. For spatial resolution measurements, the PA signals were converted to envelopes and fitted with Gaussian functions to extract lateral and axial full-width at half-maximum (FWHM). In addition, Qupath was used to conduct quantitative morphological analysis of cell nuclei area, perimeter, diameter, and aspect ratio. ImageJ was used to extract the intensity profile through line scan in the region of interest. The selected pixel width of each line is 15 pixels.

### 5.4 Deep learning-based phenotyping of epithelial wrinkle structures enabled by UV-PAM

#### 5.4.1 Dataset preparation & Pre-processing

All specimens used for deep-learning analysis were imaged during the same acquisition session using identical laser power, neutral-density filter setting, and receiver gain. Untreated and DTT-treated specimens were imaged in an interleaved order to reduce systematic acquisition-order and temporal-drift effects. Before each FOV acquisition, the axial sample position was adjusted using the same focusing procedure to place the tissue region of interest near the optical focus, and the selected focal position was maintained throughout the raster scan. The same procedure was applied to both untreated and DTT-treated specimens. Untreated wrinkle tissues comprising two biological specimens and DTT-treated wrinkle tissues comprising two biological specimens were imaged using UV-PAM. Eight FOVs were acquired for each class. Before image tiling, five FOVs from each class were assigned to the training set, while the remaining three FOVs from each class were assigned to the fixed test set. Therefore, no image patch derived from a test FOV was included in the training set. Following the FOV-level division, each FOV was tiled into 1024 × 1024-pixel patches with 50% overlap. This resulted in 1080 training patches, comprising 540 untreated and 540 DTT-treated patches, and 723 test patches, comprising 384 untreated and 339 DTT-treated patches. The training and test FOVs were obtained from the same biological specimens. An SSIM-based train–test audit detected no duplicated or near-duplicated patches, with a maximum train–test SSIM of approximately 0.73. (Supplementary Fig. S11).

#### 5.4.2 Network architecture and parameter settings

For the classification of wrinkle tissues, we employed a ResNet18-based classifier on 1024 × 1024 pixels UV-PAM images. The encoder extracted 512-dimensional global average pooled features, which were connected to a fully connected layer for binary classification between untreated and DTT-treated wrinkle tissues. A dropout layer (rate = 0.10) was applied before the classification layer, and the model was initialized with ImageNet-pretrained weights and fine-tuned on the UV-PAM dataset.

The network was optimized with the AdamW optimizer (β₁ = 0.9, β₂ = 0.999) using a learning rate of 0.0003 and weight decay of 0.0002. Training was conducted for up to 300 epochs with a mini-batch size of 32. To mitigate class imbalance, a class-weighted cross-entropy loss with label smoothing (ε = 0.05) was combined with weighted random sampling. Early stopping based on validation loss was applied with a patience of 30 epochs. Data augmentation included random 90° rotations, horizontal flips, and vertical flips. Additionally, a OneCycleLR schedule, mixed-precision training, and test-time augmentation (dihedral-8 transformations) were used to improve stability and efficiency. Five-fold cross-validation was performed at the patch level within the training set, with each model evaluated on its corresponding held-out validation fold. The five resulting ResNet18 models were subsequently combined for ensemble inference on the fixed test set by averaging their predicted class probabilities. The complete training procedure required approximately 3 h.

#### 5.4.3 Classification evaluation and explainability analysis

Classification performance was assessed using standard quantitative metrics, including accuracy, F1 score, sensitivity, precision, and specificity. These were calculated according to the following expressions:

(1) $$\mathrm{Accuracy}\;=\frac{\mathrm{TP}\;+\;\mathrm{TN}}{\mathrm{TP}\;+\;\mathrm{TN}\;+\;\mathrm{FP}\;+\;\mathrm{FN}}$$

(2) $$F1\;=\frac{2\;*\;\mathrm{Precision}\;*\;\mathrm{Recall}}{\mathrm{Precision}\;+\;\mathrm{Recall}}$$

(3) $$\mathrm{Sensitivity}\;(\mathrm{Recall})\;=\frac{\mathrm{TP}}{\mathrm{TP}\;+\;\mathrm{FN}}$$

(4) $$\mathrm{Precision}\;=\frac{\mathrm{TP}}{\mathrm{TP}\;+\;\mathrm{FP}}$$

(5) $$\mathrm{Specificity}\;=\frac{\mathrm{TN}}{\mathrm{TN}\;+\;\mathrm{FP}}$$

Where TP, TN, FP, and FN represent the numbers of true positives, true negatives, false positives, and false negatives, respectively. Final classification metrics were obtained from the ensemble predictions on the fixed test set.

To assess potential intensity-related confounding, seven global features were calculated from the normalized red channel of each image patch: mean, standard deviation, median, 95th and 99th percentiles, fraction of pixels above 0.8, and fraction of saturated pixels. Logistic-regression classifiers using either mean intensity alone or all seven features were evaluated using stratified five-fold cross-validation. Image patches were not prospectively excluded based on the presence of saturated pixels. As an additional analysis performed, pixels with normalized red-channel intensity ≥ 254/255 (defined as saturated) were systematically masked during inference, and the corresponding changes in the predicted class were recorded.

For explainability analysis, Grad-CAM++ was applied to the final convolutional block (layer4) of each ResNet18 model to identify image regions contributing to the predicted class. The resulting attribution maps were resized to the input-image resolution, normalized, and averaged across the five models to generate an ensemble-level heatmap. Occlusion-sensitivity analysis was additionally performed by masking 128 × 128-pixel regions with a stride of 128 pixels and measuring the corresponding decrease in predicted-class probability. Regions producing a greater confidence decrease were considered to have a stronger influence on the model prediction. Attribution fidelity was further assessed by comparing the confidence decrease obtained after masking the highest-attribution region with that obtained after masking a size-matched randomly selected region.

#### 5.4.4 Implementation details

All image preprocessing was performed using MATLAB R2024a (MathWorks, USA). Nuclear segmentation and morphological measurements were performed using QuPath v0.5.1 with the red detection channel, an intensity threshold of 20, a requested pixel size of 0.5 µm, and a nuclear-area range of 10–300 µm². No manual curation was applied. The classification framework was implemented in Python 3.12.7 using PyTorch 2.5.0 with CUDA 12.4. Model training and inference were performed on a Windows workstation equipped with an Intel Core i9–13900K CPU, 64 GB RAM, and an NVIDIA GeForce RTX 4090 GPU.

## CRediT authorship contribution statement

**Dong Sung Kim:** Supervision, Funding acquisition. **Byullee Park:** Writing – review & editing, Supervision, Funding acquisition, Conceptualization. **Linbin Zha:** Writing – original draft, Methodology, Investigation, Data curation. **Jangwon Yoon:** Writing – original draft, Resources, Investigation. **Santanu Misra:** Software, Data curation. **Hyunjun Kye:** Software. **Jeesu Kim:** Writing – review & editing. **Jaesung Youn:** Writing – review & editing, Supervision, Funding acquisition, Conceptualization.

## Declaration of Competing Interest

The authors declare that they have no competing interests.

## Data Availability

Data will be made available on request.
