# Supplementary material for: Label-free ultraviolet photoacoustic microscopy for multiscale structural phenotyping of epithelial morphogenesis in organoid-derived intestinal epithelial wrinkle tissue
Source: Photoacoustics. 2026 Aug 24;51:100875. doi: 10.1016/j.pacs.2026.100875 (PMC13590075; doi:10.1016/j.pacs.2026.100875)
Supplement: Supplementary file 1 [file mmc1.docx]

Supplemental Information

Label-free ultraviolet photoacoustic microscopy for multiscale structural phenotyping of epithelial morphogenesis in organoid-derived intestinal epithelial wrinkle tissue

Linbin Zha,^1†^ Jangwon Yoon,^2†^, Santanu Misra,^1†^ Hyunjun Kye,^1^ Jeesu Kim^3^, Jaesung Youn^2,4*^, Dong Sung Kim,^2,5,6*^ Byullee Park.^1*^

^1^Department of Biophysics, Department of MetaBioHealth, Department of Biopharmaceutical Convergence, Institute of Quantum Biophysics, Sungkyunkwan University, Suwon, Republic of Korea.

^2^Department of Mechanical Engineering, Pohang University of Science and Technology (POSTECH), Pohang, Republic of Korea.

^3^Department of Cogno-Mechatronics Engineering, Pusan National University, Busan 46241, Republic of Korea

^4^Department of Medical Science, School of Medicine, CHA University, Seongnam, Republic of Korea.

*^5^Graduate School of Convergence Science and Technology, Pohang University of Science and Technology (POSTECH), Pohang, Republic of Korea.*

^6^Division of Interdisciplinary Bioscience & Bioengineering, Pohang University of Science and Technology (POSTECH), Pohang, Republic of Korea

^3^[jyoun@cha.ac.kr](mailto:jyoun@cha.ac.kr), [^2,5,6^smkds@postech.ac.kr](mailto:2,5,6smkds@postech.ac.kr), [^1^byullee@skku.edu](mailto:1byullee@skku.edu)

**Supplemental Figures**

**Supplementary** **Fig. S1**. B-scan images of the targets used for spatial-resolution characterization of UV-PAM: a gold nanoparticle target for lateral-resolution measurement (left panel) and a suspended carbon-fiber target for axial-resolution measurement (right panel).

**Supplementary Fig. S2**. Ultraviolet photoacoustic microscopy (UV-PAM) of the living epithelial wrinkle tissue. The left and right panels show two regions of interest from the same live epithelial wrinkle tissue, demonstrating the capability of UV-PAM for label-free imaging of the living tissue. The scale bar is 100μm (left) and 50μm (right), respectively.

**Supplementary Fig. S3**. Depth-resolved MAPs of flat and mechanically compressed epithelial tissues. **A.** Depth-encoded MAP of flat epithelial tissue. **B.** Depth-encoded MAP of mechanically compressed epithelial wrinkle tissue. The depth-encoded MAPs were generated from the volumetric datasets shown in Fig. 3C. Compared with the flat tissue, the compressed tissue shows more spatially organized relative depth variations associated with the wrinkle morphology.

**Supplementary Fig. S4**. Workflow for quantitative analysis of wrinkle structures. **A**. Maximum amplitude projection (MAP) of human-derived lung epithelial wrinkle tissues. **B**. B-scan images of wrinkle tissues extracted along white dashed line in **A**. **C**. Magnified B-scan view of a representative single wrinkle extracted from the white box in **B**. The left valley, ridge peak, and right valley are marked. White arrows indicate consistent ridge–valley positions of wrinkle structures used for structural quantification. Wrinkle amplitude, H, was calculated as the vertical distance from the ridge peak to the local baseline connecting the two adjacent valley points. Wrinkle width, d, was defined as the lateral distance between the two valley points. The wrinkle perimeter, C, was calculated as the cumulative arc length of the extracted B-scan profile between these points. The wrinkle index was subsequently calculated as C/d.

**Supplementary Fig. S5**. Confocal microscopy (CFM) of organoid-derived intestinal epithelial wrinkle tissues. **A.** CFM of the flat epithelial tissues without external mechanical compression. **B.** CFM of wrinkle tissues with morphomechanical compression. **C.** CFM of DTT-treated wrinkle tissues. The magnified images represent cell nuclei and the distinct structural alterations of wrinkle tissues, which provide an independent reference for the structural changes observed by label-free UV-PAM.

**Supplementary Fig. S6**. Representative misclassified test patches with their corresponding Grad-CAM++ maps. **A.** False-negative cases, in which DTT-treated patches were predicted as untreated. **B.** False-positive cases, in which untreated patches were predicted as DTT-treated. The predicted probability of the assigned class is given for each case. In both panels the Grad-CAM++ maps show sparse, punctate activations at a small number of isolated locations rather than activation extending across an organized wrinkle region.

**Supplementary Fig. S7.** Red-channel intensity analysis**.** **A.** Distribution of normalized red-channel pixel intensities for untreated (n = 384) and DTT-treated (n = 339) test patches. **B.** Image-level median normalized red-channel intensity for the two groups. **C.** Image-level mean normalized red-channel intensity for the two groups. In **B** and **C**, each point represents one test patch, the box indicates the interquartile range with the central line showing the median, and the whiskers extend to the most extreme values within 1.5 times the interquartile range.

**Supplementary Fig. S8**. Complementary classification analysis using confocal microscopy (CFM) images. **A.** Average training and validation loss curves under 5-fold cross-validation, showing stable optimization of the CFM-based classifier. **B.** Summary of classification metrics, including accuracy, F1 score, precision, and recall, for distinguishing untreated and DTT-treated organoid-derived intestinal epithelial wrinkle tissues. **C.** Confusion matrix showing separation between untreated and DTT-treated samples, with sensitivity of 98.05% and specificity of 93.86%. This analysis provides a complementary comparison to UV-PAM-based computational phenotyping.

**Supplementary Fig. S9**. ROC and precision–recall analysis of the UV-PAM-based classification model. **A.** Receiver operating characteristic (ROC) curve of the UV-PAM-based classifier, with an area under the curve (AUC) of 0.962, indicating strong discriminative capacity for separating wrinkle-intact and DTT-treated wrinkle-compromised epithelial states. **B.** Precision–recall (PR) curve, with a PR-AUC of 0.960 and average precision (AP) of 0.964, further supporting the computational separability of UV-PAM-derived structural features.

**Supplementary Fig. S10**. Repeatability assessment of UV-PAM under repeated imaging of the same tissue region. **A.** Repeatability of UV-PAM of the same regions of wrinkle tissues under the same UV exposure. Scale bar, 50 μm. **B.** Quantitative analysis of nuclei area, perimeter, diameter, and aspect ratio across repeated acquisitions. Acquisition #1 was used as the reference and compared with Acquisitions #2–#4 using Welch’s two-sample t-tests. No statistically significant differences were detected for any of the evaluated morphological parameters (all p > 0.05; p = 0.074–0.941).

**Supplementary Fig. S11.** Train–test structural similarity analysis. Distribution of the maximum SSIM between each test patch and all training patches. No test patch exceeded the review threshold of 0.75 or the strict threshold of 0.95.

**Supplementary Table 1.** Descriptive comparison of nuclear morphology measured by UV-PAM and CFM

| Parameters | UV-PAM median | CFM median | Absolute difference | Relative difference |
| --- | --- | --- | --- | --- |
| Nuclei diameter/ μm | 7.37 | 7.93 | 0.56 | 7.11% |
| Nuclei area/ μm^2^ | 64.00 | 70.01 | 6.01 | 8.59% |
| Nuclei aspect ratio | 1.48 | 1.47 | 0.01 | 0.15% |
| Nuclei perimeter/ μm | 29.06 | 31.74 | 2.68 | 8.45% |

**Supplementary Table 2.** Descriptive comparison of nuclear morphology of flat and compressed tissues

| Parameters | Flat median | Compress median | Absolute difference | Relative difference |
| --- | --- | --- | --- | --- |
| Nuclei aspect ratio | 1.24 | 1.57 | 0.33 | 26.22% |
| Nuclei density/ counts mm⁻² | 5.05 × 10³ | 6.90 × 10³ | 1.85× 10³ | 36.63% |

**Supplementary Table 3.** Descriptive comparison of nuclear and wrinkle morphology of flat, untreated, and DTT-treated tissues

| Parameters | Flat median | Untreated median | DTT-treated median | Flat-untreated relative difference | Untreated-DTT-treated relative difference |
| --- | --- | --- | --- | --- | --- |
| Nuclei aspect ratio | 1.41 | 1.68 | 1.53 | 18.46% | -9.02% |
| Nuclei density/ counts mm⁻² | 5.35 × 10³ | 6.35 × 10³ | 6.1 × 10³ | 18.69% | -3.94% |
| Wrinkle amplitude/μm | / | 45 | 41 | / | -9.76% |

**Supplementary Table 4.** Summary of intensity measurements, classifier performance, and masking-based validation analyses

| **Analysis** | **Result** |
| --- | --- |
| Untreated mean red-channel intensity | 0.3457 ± 0.0870 |
| DTT-treated mean red-channel intensity | 0.3250 ± 0.0702 |
| Mean-intensity classifier accuracy | 52.15% |
| Seven-intensity-feature classifier accuracy | 58.64% |
| ResNet18 ensemble accuracy | 97.51% |
| Test patches containing saturated pixels | 586/723 |
| Prediction changes after saturation masking | 16/586 (2.7%) |
| Confidence decrease after high-attribution masking | 0.0273 ± 0.0289 |
| Confidence decrease after random-region masking | 0.0063 ± 0.0139 |

**Supplementary Table 5.** Classification performance comparison using CFM and UV-PAM

| **Imaging Modality** | **Accuracy** | **F1 Score** | **Precision** | **Recall** |
| --- | --- | --- | --- | --- |
| **CFM** | 95.85 | 95.72 | 93.50 | 98.05 |
| **UV-PAM** | **97.51** | **97.31** | **98.49** | **96.17** |

**Online Supplemental Information**

**The principle of photoacoustic microscopy**

Photoacoustic microscopy is an emerging imaging technology based on photoacoustic effect. When the short-pulse laser irradiates the tissue, the locally absorbed light is converted into the heat. The subsequent thermoelastic expansion generates the photoacoustic wave. The initial pressure of the generated photoacoustic wave can be expressed as follows[1, 2]:

$P_{0}=\Gamma\eta_{th}\mu_{a}F$. (S1)

Here, $\Gamma$ represents the Grueneisen parameter. $\eta_{th}$ denotes the heat conversion which the absorbed light is transformed into thermal energy. $\mu_{a}$ is the coefficient of optical absorption. $F$ indicates the optical fluence.

**References**

1. Yang JG, Choi SW, Kim JW, Park BL, Kim CH, Recent advances in deep-learning-enhanced photoacoustic imaging. Adv. Photon. Nexus 2023;2(5):054001.

2. Choi WS, Park BL, Choi SW, Oh DH, Kim JB, Kim CH. Recent advances in contrast-enhanced photoacoustic imaging: overcoming the physical and practical challenges. Chem. Rev. 2023;123:7379-7419.
